# U.S. active school travel in 2017: Prevalence and correlates

**DOI:** 10.1016/j.pmedr.2019.101024

**Published:** 2019-12-10

**Authors:** Eleftheria Kontou, Noreen C. McDonald, Kristen Brookshire, Nancy C. Pullen-Seufert, Seth LaJeunesse

**Affiliations:** a Department of City and Regional Planning, University of North Carolina at Chapel Hill, New East Building, CB3140, Chapel Hill NC 27599, USA; b The University of North Carolina Highway Safety Research Center, 730 M.L.K. Jr Blvd, Chapel Hill NC 27514, USA

**Keywords:** ATS, active transportation to school, CBSA, Core-Based Statistical Area, NHTS, National Highway Travel Survey, SRTS, Safe Routes to School, Active transportation, Bicycling, Children, Mode choice, School travel, Walking

## Abstract

Active transportation to school (ATS), denoting walking and biking, is crucial to promote physical activity for youth. This study uses data from the 2017 National Household Travel Survey (NHTS) to report on the most recent and nationally representative school transportation patterns. Binary logit modeling determines significant factors associated with school travel mode choices. Spatial differences on school mode choices across the US are explored. In 2017 9.6% of the students of 5–17 years old usually walked and 1.1% biked to school. For students who usually walk to school, 77.5% of their school trips were less than one mile and, among usual bikers to school, 82.8% of trips were less than two miles. Student rates of walking to school decreased as the distance to school increased and biking rates peaked when distance to school was between 0.5 and 1 miles. When distance to school was <0.5 miles, walking was the most common mode for urban and rural regions. When the trip was shorter than or equal to one mile, factors such child's school grade, household vehicles per driver, and household income were associated with the decision to walk or bike to school. Other demographic characteristics like race and gender were not significantly related to ATS. While comparison across NHTS years should be viewed with caution due to changes in survey methodology, the decline of ATS rates indicate that more effective and higher reaching efforts for local, regional, and national interventions should be prioritized.

## 1 Introduction

Nearly 52 million American children and adolescents travel to school every day (US Census Bureau, 2016). Increasing the proportion of these students that walk or bike to school is a national health goal (Community Preventive Services Task Force, 2018). Active transportation to school (ATS) is associated with building healthy activity and eating habits and contributing to leading physical active lives (Cooper et al., 2005, Cooper et al., 2003, Madsen et al., 2015). The prevalence of ATS for students in grades K-8 was almost 48% in the 1970s but declined to 13% by 2009 in the United States (McDonald et al., 2011) with similar downward trends observed in certain Canadian regions, the United Kingdom, and Australian regions (Buliung et al., 2009, Timperio et al., 2006, Tudor-Locke et al., 2001).

Surveillance of ATS is critical to measuring movement toward achieving national health objectives. The National Household Travel Survey (NHTS), conducted by the US Department of Transportation, is the only source of national-level surveillance data on school travel (USDOT, 2017). Our study uses the 2017 NHTS to report on the prevalence of ATS and disaggregate ATS shares by characteristics such as distance to school or urban/rural residence environment classification of a student’s residence. Previous research found these characteristics to be correlated with ATS (Buttazzoni et al., 2018, McDonald, 2012, Panter et al., 2010). Our analysis also reports on trip, individual, and household correlates of ATS using binary logit models.

The study’s objective is twofold: (1) document the prevalence of ATS using the most recently available national surveillance data and (2) uncover demographic and geographic factors associated with ATS. A current understanding of school travel mode share and correlates for ATS can help federal agencies and their partners at the state and local level track progress toward achieving health goals and the opportunities for tailoring interventions, such as Safe Routes to School (SRTS) programs. SRTS aims to increase the safety and prevalence of ATS through engineering, education, enforcement, and encouragement efforts (McDonald et al., 2014). Previous research found that SRTS interventions may improve ATS shares; however, effectiveness varies across studies (Boarnet et al., 2005a, Buttazzoni et al., 2018, Chillon et al., 2011, Larouche et al., 2018, Villa-González et al., 2018). Findings from the NHTS also provide a benchmark for local communities as they examine their own school travel patterns to inform decisions about school location, assignment, and transportation.

## 2 Methods

### 2.1 Data

The NHTS collects data on Americans’ travel patterns. It has been conducted at irregular intervals since 1969 (USDOT, 2017). The survey is the primary source of surveillance data on US school travel. The survey collects data on households, individuals, and all travel in a household on a randomly assigned survey day. The latest survey dataset, collected in 2017 and analyzed between August and October 2018, includes variables that provide insights on youth school travel, such as distance and mode to school. The 2017 NHTS utilized a two-stage, mixed mode data collection process, which involved a mailout/mail-back recruitment stage and a primarily web-based system for retrieving data about travel on the assigned survey day (Westat, 2017). The survey design used address-based sampling (ABS) to improve coverage; previous surveys (1995, 2001, 2009) used random-digit dialing (RDD) of landlines to recruit respondents (Westat, 2018). Participants were offered a multi-stage incentive for continued participation in the survey (initial recruitment $2, continued participation $5 for each travel log retrieval and $20 when all members completed the travel inventories) (Couper et al., 2016). Travel distances were estimated from Google Maps API shortest-path route per mode rather than relying on respondent self-report, which was the case for previous years’ data collection (Mcguckin et al., 2018). Note that Dessing et al., 2016, Zhu and Levinson, 2015 pinpointed that the shortest route is not always the actual preferred route. The 2017 survey required all household members above 5 years old to complete a retrieval interview in order to consider it a complete submission (Roth et al., 2017). For household members <16 years old, an adult proxy documented their travel (US Department of Transportation, 2017). The weighted person-level NHTS response rate is 15.6% (Westat, 2018).

### 2.2 Measures of school travel

The 2017 NHTS includes two school travel outcome measures: usual travel mode to and from school and travel mode to and from school on the survey day. Usual travel mode to school is based on the question “*How do you usually get to school?*” and was collected for all student subjects and analyzed in this study. Data on the survey-day travel mode to and from school was collected for individuals that reported a trip purpose of “ATTEND SCHOOL AS A STUDENT” and who attended “PUBLIC OR PRIVATE SCHOOL” when asked “*What type of school do you attend*” (only refers to subjects between 5 and 17 years old). Since usual mode and travel-day mode to school are not always the same, examining both these measures helps evaluating the reliability of our outcomes.

### 2.3 Descriptive analysis

Descriptive statistics provide national averages on school travel mode use, including overall and by distance to school (including categories <0.25, [0.25, 0.5), [0.5, 1), [1, 2], >2 miles), urban/rural classification, and age/school grade (elementary – ages [5, 11], middle - ages [12,14], high – ages [15, 17]). Weighting factors, based on “calculating the inverse of the selection probability for each sampled address as a base weight, adjusting the base weights for eligibility and nonresponse, and poststratifying the adjusted weights to reliable external source data, such as Census data” (Roth et al., 2017), are readily available with the NHTS dataset to calculate nationally representative estimates from the 2017 NHTS sample. Regional variation is documented by reporting ATS prevalence for Core-Based Statistical Areas (CBSAs) with youth unweighted sample sizes above 200.

### 2.4 Binary logit model

Binary logit models focused on trips less than or equal to one mile and estimate the probability of usually reaching school by active transportation modes (walking or biking) versus driving, taking the school bus, or utilizing another mode. The binary logit model focus on trips <1 mile because walking and biking could be a realistic part of the choice set for this distance to school (Gropp et al., 2012, McDonald et al., 2011). All the independent covariates are entered in the binary logit model. Survey weights are not applied for the model because the data are not stratified on the outcome measure of travel mode and, under such conditions, weights use is not recommended in logit model estimation (Ben-Akiva and Lerman, 1985). The statistical analysis described here was conducted using Python 2.7.15. The paper presents primarily results for the usual ATS to school dependent variable; descriptive statistics for ATS use on the survey-day are available mostly in the Appendix for interested readers.

### 2.5 Sample size

The 2017 NHTS includes demographic and travel information on 35,197 (N = 58,576,741) children and adolescents between the ages of 5 and 17 (inclusive). For the analysis of usual travel mode to school, respondents are excluded from the analysis if there is missing information on usual school travel mode (n = 4,372), distance to school (n = 82), race and ethnicity (n = 194), household income (n = 447), and sex (n = 38). The final sample size for descriptive statistics analysis of usual school travel mode was 30,064 (N = 48,339,487). The sample size for the binary logit model was 5,732 (N = 9,551,563) for the usual mode and 3,461 (N = 2,090,776) for the survey-day travel due to restricting the analysis to individuals living within one mile of school and missing data on model variables.

## 3 Results

American youth traveled 43.8 billion person-miles and conducted approximately 9.56 billion travel day person-trips one-way to school in 2017. For youth between 5 and 17 years old, trips going to school accounted for 8.9% of their annual person-miles and 17.1% of their annual person-trips. The median distance to school in 2017 is 2.7 miles (elementary students median is 2.1 miles, middle school students 3.2 miles, and high school students 3.6 miles to school) and its duration on average 18 min for all modes of choice.

Table 1 reports unweighted summary statistics for several youth and household demographic characteristics, for the usual and the survey-day travel samples.

**Table 1:** 2017 NHTS summary statistics for school travel, unweighted %

	Usual Travel Mode to School	Survey-day Travel Mode to School
**Sample Size**	30,064	17,766
**Age mean (standard deviation)**	11.1 (3.6)	11.5 (3.9)
**Avg. distance to school (mi)** **[interquartile range (mi)]**	6.9 [1.3, 5.6]	4.8 [1.2, 5.4]
**Avg. minutes to school (min)** **[interquartile range (min)]**	n.a.	17.9 [8, 25]
**School Level**		
Elementary (5–11 years old)	52.7%	48.9%
Middle (12–14 years old)	24.3%	23.1%
High (15–17 years old)	23.0%	28.0%
**Gender (youth respondent)**		
Female	48.7%	48.8%
Male	51.3%	51.2%
**Race/Ethnicity**		
Non-Hispanic white	62.5%	68.4%
Non-Hispanic black	8.8%	8.9%
Hispanic/Latino	16.0%	12.1%
Other	12.7%	10.6%
**Household Income Levels**		
0–35,000	19.4%	19.0%
35,000–75,000	24.6%	24.5%
75,000–125,000	27.9%	27.6%
>125,000	28.1%	28.9%
**Household Vehicle Ownership**		
Zero Vehicle	2.1%	1.9%
1 Vehicle	16.5%	16.0%
2 Vehicles	46.4%	44.5%
≥3 Vehicles	35.1%	37.5%
**Home Ownership**		
Own	75.8%	76.0%
Rent	23.5%	23.2%
Other/Missing	0.7%	0.9%
Residence’s Census Block Population Density		
0–500 persons per sq. mile	27.1%	26.9%
500–1,000 persons per sq. mile	9.3%	9.2%
1,000–3,999 persons per sq. mile	33.2%	33.8%
4,000–9,999 persons per sq. mile	24.1%	24.0%
≥10,000 persons per sq. mile	6.3%	6.0%
Missing	0.6%	0.06%
**Urban Environment**	77.9%	79.5%
**Residing < 1 mile from School**	19.1%	19.5%

### 3.1 Travel mode

In 2017, 9.6% (95%CI: 8.1–11.0) of students usually walked and 1.1% (95%CI: 0.7–1.6) usually biked to school; 50.2% (95%CI: 47.9–53.0) usually reached school by car, as shown in Table 2. School bus usage in 2017 reached 36.6% (95%CI: 34.1–38.3). ATS differs across school grade levels; biking to school levels are the highest for middle schoolers and walking levels are the highest for the elementary grades.

**Table 2:** Usual mode to school by school grade (elementary, middle, high, and all), weighted %

Mode	Elementary (5–11 yo)	Middle (12–14 yo)	High (15–17 yo)	All (5–17 yo)
Auto	51.6	41.8	56.2	50.2
Walk	10.0	9.9	8.0	9.6
Bike	0.9	1.8	0.8	1.1
School Bus	36.4	43.4	29.4	36.6
Other	1.1	3.1	5.6	2.5
**Total**	100	100	100	100

Note: yo stands for years old.

**Fig. 1:**
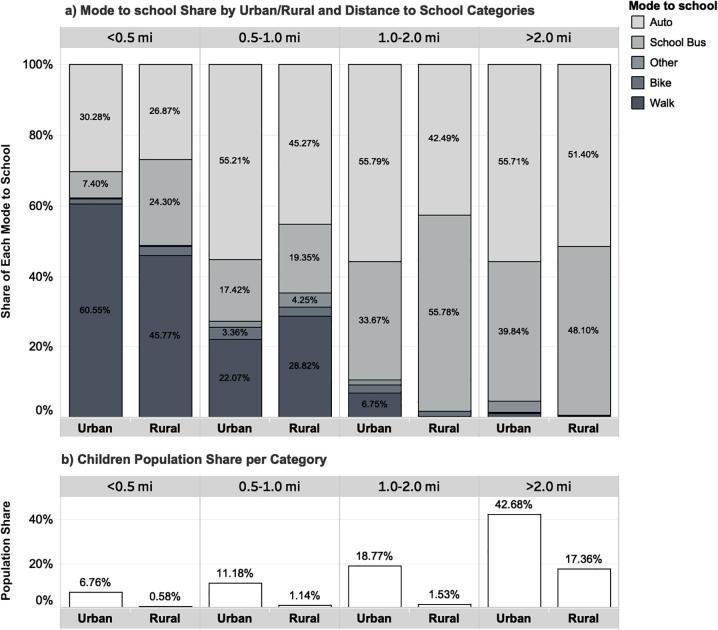
Distribution of usual mode choice across urban/rural and distance to school categories, weighted %

For students who usually walk to school, 77.5% of their reported trips were less than one mile; 16.4% reported trips between one and two miles; and 6.1% reported trips longer than two miles. Among bikers to school, 82.8% of trips were less than two miles. Fig. 1 reports modal prevalence disaggregated by urban/rural classification and distance to school. The urban/rural classification is based on the US Census definitions of urbanized areas and urban clusters (U.S. Federal Highway Administration, 2019a). When distance to school is less than or equal to 0.5 miles, the greatest share of trips to school are conducted on foot in both urban and rural areas. As distance to school increases from 0.5 to 1 mile, the portion of travel to school by bike increases in urban and decreases in rural environments. For almost all distance to school categories apart from the rural category that corresponds to <0.5 miles distance to school, which reflects a limited population share, the prevalence of school bus to travel to school is greater in rural compared to urban environments.

The prevalence of ATS varies spatially. The results presented in Table 3 are the only ones spatially constrained to each CBSA, which consists of counties that are associated with at least one urban cluster (≥10,000 population). The proportion of students that usually walk to school in the Los Angeles CBSA is 17.3% (95% CI: 14.7–20.21); 15.5% (95% CI:12.9–18.6) in the San Diego Area; 15.4% (95% CI: 12.6–18.7) in the San Francisco Bay Area; and 11.40% (95% CI: 9.8–13.2) in the New York City Area. Biking rates to school higher than national averages are observed in San Francisco CA CBSA (5.32% (95% CI:3.7–7.6)) and Houston TX (2.10% (95% CI:1.9–3.7)).

**Table 3:** Variation of usual AST percentage across CBSAs in the U.S., weighted %

Core-Based Statistical Area (CBSA)	Sample Size [5–17 age sample (5–17 age population)]	Walk %	Bike %
Atlanta-Sandy Springs-Roswell, GA	761 (935,798)	4.36	0.26
Chicago-Naperville-Elgin, IL-IN-WI	244 (1,327,359)	10.67	2.37
Dallas-Fort Worth-Arlington, TX	2,518 (1,256,491)	7.58	1.59
Houston-The Woodlands-Sugar Land, TX	1,400 (1,134,623)	5.58	2.69
Los Angeles-Long Beach-Anaheim, CA	719 (1,688,869)	17.32	1.75
New York-Newark- Jersey City, NY-NJ-PA	1,350 (2,709,044)	11.40	0.21
San Diego-Carlsbad, CA	595 (554,096)	15.50	1.47
San Francisco-Oakland-Hayward, CA	526 (744,379)	15.41	5.32

### 3.2 Binary logit model

**Fig. 2:**
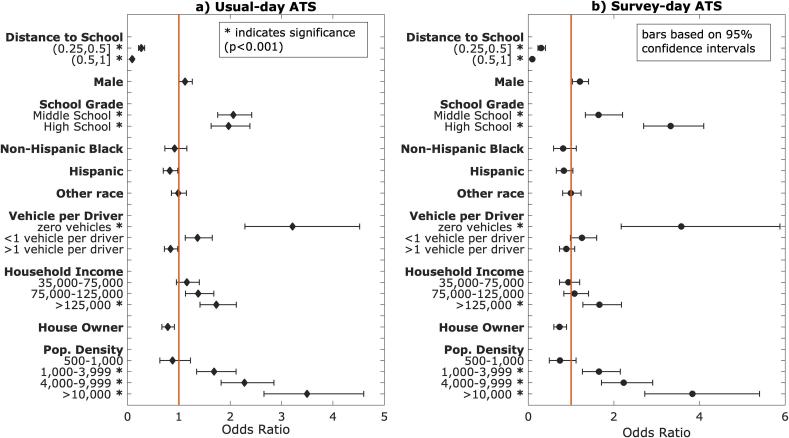
Factors associated with probability of usual and survey-day ATS for trips less than or equal to one mile. Reference categories of the independent variables included are indicated as follows: Distance to School [0,0.25], Gender female, School Grade elementary, Vehicle per Driver 1veh/driver, Household Income ≤35,000, Pop. Density ≤500.

Binary logit models (usual and survey-day ATS vs. no ATS) show associations between travel mode and socio-demographics and location characteristics for youth living within one mile from the school they attend. All variables presented in Fig. 2 were included in the model runs. Distance to school and population density had the strongest effect on walking and biking to school, with the odds of walking/biking to school increasing as distance to school decreases and population density increases. All significant covariates at a 0.05 significance level are denoted with an asterisk in the y axis of the a) and b) subgraphs of Fig. 2. The odds of ATS decrease as household income decreases and vehicle ownership rate (vehicles per driver) increases.

## 4 Discussion

In 2017, dropping off and picking up school-age children from school corresponded to 1.49% of the total annual vehicle miles traveled in the US. Driving students to school or youth driving themselves there during the morning peak (7:00am to 9:00am) corresponded to 10% of total vehicle trips and to approximately 8% of the total vehicle miles traveled. Approximately one in ten American students usually walked or biked to school; the majority used private automobiles or school buses to access their educational facility. These results are aligned with Federal Highway Administration’s brief analysis (U.S. Federal Highway Administration, 2019b).

Comparison of K-8 ATS prevalence in 2017 to reports from the 2009 NHTS showed modest declines (McDonald et al., 2011), from 12.7% in 2009 to 11.1% in 2017. However, significant changes in NHTS survey methodology between 2009 and 2017, such as moving to an address-based sample from a landline-based sample, make it difficult to assess change over time from simply comparing prevalence. Other critical survey differences include the reduction of travel mode options offered on the survey in 2017 from 35 to 20 and real-time geocoding of origins and destinations compared to self-reported distances in 2009 which resulted in 10% shorter trips accounting for all trips (not only active), compared to previous years NHTS outcomes (US Department of Transportation, 2017). It is difficult to determine whether distance self-reporting or shortest path geocoding over- or under-estimated distance. For example, shortest path is not always the path taken by travelers and paths might be determined by weather, temperature, time of day, experience, and other characteristics (Zhu and Levinson, 2015). Even though, compared to previous years, the prevalence of ATS is lower (McDonald, 2006, McDonald et al., 2011), a recent study that focused on metropolitan areas (Le et al., 2019) found a 2–6% increase on total urban bicycle and walking volumes, leveraging non-motorized travel monitoring data.

Using 2017 NHTS, for the nationally representative sample aged 5–17 years old and residing a mile or less from school, 40.31% walk or bike to school frequently – a reduced share compared to a 47.9% share by Martin et al. analysis (Martin et al., 2007) of the 2004 Youth Media Campaign Longitudinal Survey. Under the assumption that distance to school below 2 miles (sample size: 40.35%) is bikeable, 2.18% bike to school frequently. Observed ATS reductions are concerning, highlighting the need for investments in research and interventions that can improve the safety and comfort of walking or biking to school, such as SRTS infrastructure improvement projects (Boarnet et al., 2005b).

**Appendix Fig. 1:**
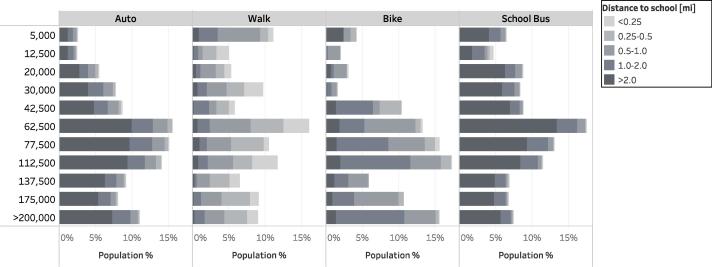
Distribution across income categories for each mode to school use on usual day, weighted %.

Correlations between distance to school, socio-demographics, and ATS remained consistent from 2009 (McDonald et al., 2011) to 2017. The binary logit modeling identified significant factors related to ATS, showcasing the criticality of distance to school and residential population density to such decisions, well-aligned with relevant literature findings (McDonald, 2008, Rothman et al., 2018). Increased ATS odds for higher income households was also the outcome of a 2009 NHTS binary logit model (McDonald et al., 2011). Appendix Fig 1 portrays for each mode use the population distribution across income categories, confirming the modeling result conclusion related to household income. Further research is needed to show whether more affluent households have greater access to safer bicycling and walking infrastructure and whether such ATS income-based disparities parallel income-based health disparities.

The difference in ATS shares by population density is potentially related to built environment characteristics that promote safe walking and biking, such as sidewalks and bike lanes respectively (Davison and Lawson, 2007). Geospatial variations (Mitra and Buliung, 2012, Wong et al., 2011) across CBSAs compared to the national-level ATS shares highlight the importance of conducting localized studies in order to explore ATS barriers and understand the effectiveness of intervention programs like SRTS. Spatial ATS variation may hint at different culture, climate, and weather impacts (Sirard et al., 2005). Spatial differences may be also attributed to population density and socio-demographics characterizing those regions (Davison and Lawson, 2007, Wolfe and McDonald, 2016), as well as school transport policies and interventions success to induce ATS (Buttazzoni et al., 2018, Larouche et al., 2018). Differences between usual and survey-day ATS could be also dependent on environmental phenomena (Sirard et al., 2005). Survey-day automobile mode shares were higher than the usual-day ones, suggesting that car school transportation is the most reliable back-up option.

Socio-demographic factors such as gender, race, and ethnicity did not critically affect the ATS choice. Our study found that female youth used active transportation less often than males, similar to McDonald, 2012, even though the difference is modest and significant only at a 0.1 level. Race and ethnicity were not associated with ATS, contrary to prior evidence (Madsen et al., 2015, McDonald et al., 2011, Wolfe and McDonald, 2016). Caregivers’ safety concerns and travel attitudes were not examined here, but were impactful in previous ATS research (Martin et al., 2007, Wolfe and McDonald, 2016). The odds of ATS here were reduced with home-ownership, similar to 2009 choices effects (McDonald et al., 2011). Home and automobile ownership signify household indicators that are related with healthy habits for American youth such as ATS, well-aligned with existing work (McDonald, 2007). Relationships between income, population density, and the neighborhood’s built environment could be also uncovering school access equity concerns (Panter et al., 2010) and residential self-selection (Cao et al., 2009).

## 5 Conclusion

Based on the 2017 National Household Travel Survey Data analyses, <10% usually walked to school and approximately 1.1% usually biked to school. More than three fourths of the usual day walking trips to school were less than one mile, when biking rates reach peak for distance to school between 0.5 and 1 mile. The odds of ATS (for distances to school ≤1 mile) increase for youth with residences closer to schools, zero and low vehicle ownership, residing in areas with greater population density, higher household income, and of higher school level. Regional and local studies should be pursued when evaluating ATS shares and interventions, due to significant spatial differences compared to national-level averages.

Given the potential of ATS to promote physical activity (Larouche et al., 2018), future research may need to closer evaluate outcomes of interventions after targeting the most vulnerable youth segments in order to observe tangible walking and biking share improvements. We observe income disparities in ATS, which might hint at health disparities and highlight the need for ATS interventions in lower-income areas. Results presented here underscore the importance of tracking ATS shares through surveillance and modeling to uncover critical factors that affect such choices through time, to enable national-level comparisons and monitoring. The role of the federal government as a data collector for national ATS shares and promoter of best ATS practices should be elevated, assisting local stakeholders with identifying appropriate interventions to improve ATS rates.

## 6 Declarations

**Ethics Approval and consent to participate**

Not applicable.

**Consent for publication**

Not applicable.

**Availability of data and material**

The datasets used and/or analyzed during the current study are available from the corresponding author on reasonable request.

## Funding

The authors were supported through two US Department of Transportation sources: a cooperative agreement from Federal Highway Administration (FHWA) and funds from the Southeastern Transportation Research, Innovation, Development and Education Center, the University-based Transportation Center for the southeastern region through the Office of the Assistant Secretary of Technology.

**Authors' contributions**

EK analyzed and interpreted the 2017 National Household Travel Survey data and was a major contributor in writing the manuscript. NM acquired funding and was a major contributor in writing the manuscript. KB, NPS, and SL acquired funding and were major contributors in editing and critically reviewing the manuscript. All authors read and approved the final manuscript.

## Declaration of Competing Interest

The authors declare that they have no known competing financial interests or personal relationships that could have appeared to influence the work reported in this paper.

## Figures and Tables

**Table:** 

	Distance to school	HH Income	House Owner	Other race	Non-Hispanic Black	Vehicles per Driver	Population Density	ATS
Distance to school	1	0.0399	0.0761	−0.0603	0.0193	0.0657	−0.2714	−0.495
HH Income	0.0399	1	0.439	−0.0408	−0.2288	0.2004	0.0061	−0.0089
House Owner	0.0761	0.439	1	−0.0558	−0.2385	0.2231	−0.171	−0.0771
Other race	−0.0603	−0.0408	−0.0558	1	−0.148	−0.0433	0.1493	0.0476
Non-Hispanic Black	0.0193	−0.2288	−0.2385	−0.148	1	−0.1017	0.069	−0.0041
Vehicles per Driver	0.0657	0.2004	0.2231	−0.0433	−0.1017	1	−0.1547	−0.0821
Population Density	−0.2714	0.0061	−0.171	0.1493	0.069	−0.1547	1	0.1933
ATS	−0.495	−0.0089	−0.0771	0.0476	−0.0041	−0.0821	0.1933	1

**Table:** 

Mode	Elementary (5–11)	Middle (12–14)	High (15–17)	All (5–17)
Auto	54.0	48.0	62.5	55.0
Walk	9.6	9.4	11.6	10.1
Bike	0.6	1.7	0.7	0.9
School Bus	34.6	38.7	10.3	31.5
Other	1.2	2.2	5.0	2.5
**Total**	100	100	100	100

**Table:** 

Miles to School	Auto	Walk	Bike	School Bus	Other	Total
<0.25	11.9	81.9	0.4	5.1	0.7	100
0.25–0.5	32.0	58.6	1.1	7.9	0.4	100
0.5–1.0	55.5	22.8	2.7	17.5	1.5	100
1.0–2.0	58.3	5.9	1.0	32.7	2.1	100
>2.0	58.0	0.8	0.4	37.7	3.1	100

**Table:** 

	Usual Day Travel (≤1mile)		
Independent Variable	OR	p-value	95% CI
Distance to school			
0–0.25	reference		
0.25–0.5	0.266	<0.0001*	0.213–0.332
0.5–1	0.092	<0.0001*	0.074–0.114
**Male**	1.121	0.0591	0.995–1.262
**School grade/age**			
Elementary (5–11)	reference		
Middle (12–14)	2.06	<0.0001*	1.756–2.419
High (15–17)	1.969	<0.0001*	1.626–2.385
**Non-Hispanic Black**	0.915	0.4537	0.725–1.153
**Hispanic**	0.824	0.0315	0.695–0.977
**Other race(non-white, non-black)**	0.989	0.8899	0.854–1.147
**Vehicles per Driver**			
Zeros Vehicles	3.214	<0.0001*	2.285–4.520
<1 vehicle per driver	1.361	0.0015	1.123–1.649
1 vehicle per driver	reference		
>1 vehicle per driver	0.839	0.0265	0.718–0.979
**Household Income**			
0–35,000	reference		
35,000–75,000	1.155	0.1388	0.954–1.398
75,000–125,000	1.377	0.0017	1.128–1.682
>125000	1.729	<0.0001*	1.410–2.120
**House Owner**	0.783	0.0019	0.671–0.914
**Population Density**			
0–500	reference		
500–1,000	0.878	0.445	0.629–1.225
1,000–3,999	1.687	<0.0001*	1.346–2.114
4,000–9,999	2.278	<0.0001*	1.821–2.849
≥10,000	3.496	<0.0001*	2.659–4.600
**Observations**	5,732		
**Log Likelihood**	−3,242.09		
**Pseudo R-square**	0.1331		

Note: * p < 0.001 (indicating significance); OR, odds ratio; CI, confidence interval.

**Table:** 

	Survey-Day Travel (≤1 miles)		
Independent Variable	OR	p-value	95% CI
Distance to school			
0–0.25	reference		
0.25–0.5	0.300	<0.0001*	0.226–0.398
0.5–1	0.091	<0.0001*	0.069–0.120
**Male**	1.204	0.0193	1.030–1.405
**School grade/age**			
Elementary (5–11)	reference		
Middle (12–14)	1.643	<0.0001*	1.334–2.203
High (15–17)	3.325	<0.0001*	2.696–4.099
**Non-Hispanic Black**	0.813	0.2101	0.589–1.123
**Hispanic**	0.827	0.1082	0.657–1.042
**Other race (non-white, non-black)**	0.995	0.9662	0.805–1.231
**Vehicles per Driver**			
Zeros Vehicles	3.573	<0.0001*	2.171–5.879
<1 vehicle per driver	1.254	0.0674	0.984–1.597
1 vehicle per driver	reference		
>1 vehicle per driver	0.888	0.2431	0.729–1.083
**Household Income**			
0–35,000	reference		
35,000–75,000	0.933	0.5929	0.727–1.200
75,000–125,000	1.081	0.5649	0.829–1.409
>125000	1.664	0.0002*	1.273–2.176
**House Owner**	0.730	0.0024	0.597–0.895
**Population Density**			
0–500	reference		
500–1,000	0.7416	0.1532	0.492–1.118
1,000–3,999	1.6493	0.0002*	1.264–2.151
4,000–9,999	2.2278	<0.0001*	1.707–2.907
≥10,000	3.8369	<0.0001*	2.724–5.404
**Observations**	3,461		
**Log Likelihood**	−1,911.73		
**Pseudo R-square**	0.1618		

Note: * p < 0.001 (indicating significance); OR, odds ratio; CI, confidence interval.

## Appendix

See Fig. A1

Appendix Table 1. Variables Correlations.For the survey-day ATS analysis, 18,164 youth reported a school trip. Individuals were excluded if data was missing information on travel mode (n = 0), travel distance to school (n = 22), race and ethnicity (n = 94), household income (n = 282), and sex (n = 0). The resulting sample was 17,766 with information on the survey-day school travel mode.

Appendix Table 2. Mode to school on survey-day by school grade, weighted %.Appendix Table 3. Percent of students that walk, bike, or use other mode to school on survey-day for each distance to school category, weighted %Comparing usual mode choices to survey day mode choices, higher rates of automobile use to reach school are observed, particularly for residence distances further away from school (>1mile). Survey day biking rates are lower compared to the usual day.

Appendix Table 4. Factors associated with the probability of **usual day ATS** for trips less than or equal to one mile (unweighted sample)

Appendix Table 5. Factors associated with the probability of **survey-day ATS** for trips less than or equal to 1 mile (unweighted sample).
